# The Integrative Conjugative Element *clc* (ICEclc) of *Pseudomonas aeruginosa* JB2

**DOI:** 10.3389/fmicb.2018.01532

**Published:** 2018-07-11

**Authors:** Chioma C. Obi, Shivangi Vayla, Vidya de Gannes, Mark E. Berres, Jason Walker, Derek Pavelec, Joshua Hyman, William J. Hickey

**Affiliations:** ^1^Department of Biological Sciences, Bells University of Technology, Ota, Nigeria; ^2^Department of Soil Science, University of Wisconsin-Madison, Madison, WI, United States; ^3^Department of Food Production, University of the West Indies, St. Augustine, Trinidad and Tobago; ^4^Biotechnology Center, University of Wisconsin-Madison, Madison, WI, United States

**Keywords:** integrative conjugative element (ICE), ICEclc, biodegradation, xenobiotic metabolism, PCBs, chlorobenzoates, *Pseudomonas aeruginosa*

## Abstract

Integrative conjugative elements (ICE) are a diverse group of chromosomally integrated, self-transmissible mobile genetic elements (MGE) that are active in shaping the functions of bacteria and bacterial communities. Each type of ICE carries a characteristic set of core genes encoding functions essential for maintenance and self-transmission, and cargo genes that endow on hosts phenotypes beneficial for niche adaptation. An important area to which ICE can contribute beneficial functions is the biodegradation of xenobiotic compounds. In the biodegradation realm, the best-characterized ICE is ICEclc, which carries cargo genes encoding for *ortho*-cleavage of chlorocatechols (*clc* genes) and aminophenol metabolism (*amn* genes). The element was originally identified in the 3-chlorobenzoate-degrader *Pseudomonas knackmussii* B13, and the closest relative is a nearly identical element in *Burkholderia xenovorans* LB400 (designated ICEclc-B13 and ICEclc-LB400, respectively). In the present report, genome sequencing of the *o*-chlorobenzoate degrader *Pseudomonas aeruginosa* JB2 was used to identify a new member of the ICEclc family, ICEclc-JB2. The cargo of ICEclc-JB2 differs from that of ICEclc-B13 and ICEclc-LB400 in consisting of a unique combination of genes that encode for the utilization of *o*-halobenzoates and *o*-hydroxybenzoate as growth substrates (*ohb* genes and *hyb* genes, respectively) and which are duplicated in a tandem repeat. Also, ICEclc-JB2 lacks an operon of regulatory genes (*tciR-marR-mfsR*) that is present in the other two ICEclc, and which controls excision from the host. Thus, the mechanisms regulating intracellular behavior of ICEclc-JB2 may differ from that of its close relatives. The entire tandem repeat in ICEclc-JB2 can excise independently from the element in a process apparently involving transposases/insertion sequence associated with the repeats. Excision of the repeats removes important niche adaptation genes from ICEclc-JB2, rendering it less beneficial to the host. However, the reduced version of ICEclc-JB2 could now acquire new genes that might be beneficial to a future host and, consequently, to the survival of ICEclc-JB2. Collectively, the present identification and characterization of ICEclc-JB2 provides insights into roles of MGE in bacterial niche adaptation and the evolution of catabolic pathways for biodegradation of xenobiotic compounds.

## Introduction

Integrative conjugative elements (ICE) are a diverse group of chromosomally integrated mobile genetic elements (MGE) that are active in shaping the behavior of bacteria and bacterial communities (Wozniak and Waldor, 2010). ICE are self-transmissible from host chromosomes, and each type of ICE carries a characteristic set of core genes that encode for its excision, circularization, conjugative transfer and site-specific integration in a new host (Johnson and Grossman, 2015; Banuelos-Vazquez et al., 2017; Cury et al., 2017; Delavat et al., 2017). The other components of ICE are the cargo genes, which encode functions affecting bacterial life styles and niche adaptation. ICEs have been most extensively studied with respect to their roles in conferring virulence factors and/or resistance to antimicrobial compounds (Chowdhury et al., 2016; Clawson et al., 2016; Leon-Sampedro et al., 2016; Olaitan et al., 2016; Bie et al., 2017; Chuzeville et al., 2017; Ryan et al., 2017; Sugimoto et al., 2017; Vanneste, 2017; Zhou et al., 2017). But, the spectrum of ICE-encoded adaptation functions is broad and also includes resistance to heavy metals (Colombi et al., 2017; Harmer et al., 2017), rhizobial nodulation functions (Ling et al., 2016), biofilm formation characteristics (Wang et al., 2017) and components of metabolic pathways (Gaillard et al., 2006; Zamarro et al., 2016; Suenaga et al., 2017).

Biodegradation of xenobiotic compounds is a key environmental service of bacterial communities, and MGE are well-established as playing a central role in the evolution of metabolic capacity essential for these activities (Top et al., 2002; Top and Springael, 2003; Diaz, 2004; Shintani et al., 2010). While initial work exploring MGE centered largely on plasmids, the advent of genome sequencing has revealed the role of ICE and other types of genomic islands (van der Meer and Sentchilo, 2003; Gaillard et al., 2006; Hickey et al., 2012; Chong et al., 2014; Pathak et al., 2016; Zamarro et al., 2016; Suenaga et al., 2017).

In the biodegradation realm, the best-characterized ICE is termed ICEclc and was originally identified in the 3-chlorobenzoate-degrader *Pseudomonas knackmussii* B13 (Gaillard et al., 2006). Integration of ICEclc is mediated by an integrase that is located a terminus of the element (IntB13). Within ICEclc, genes encoding core functions (maintenance and stability of the element) are segregated to one side while those encoding biodegradation functions are grouped to the other (Gaillard et al., 2006). Biodegradation functions encoded by ICEclc include the pathway for *ortho*-cleavage of chlorocatechols (*clc* genes) and *amp* genes encoding aminophenol metabolism (Gaillard et al., 2006). A number of other ICE and genomic islands are related to ICEclc by synteny in core functions (Gaillard et al., 2006), and one of these that occurs in *Burkholderia xenovorans* LB400 (currently *Paraburkholderia xenovorans* LB400, (Sawana et al., 2014)) is identical except for an additional *ca*. 1 kb of cargo and one extra gene in core region; this element is hereafter referred to as ICEclc-LB400 to distinguish it from the original ICEclc (hereafter referred to as ICEclc-B13).

Polychlorinated biphenyls (PCBs) are an important group of environmental contaminants for which biodegradation is a primary pathway of removal (Field and Sierra-Alvarez, 2008). However, bacteria that effect these transformations typically do so by cometabolism (i.e., growth on biphenyl), in part because they cannot utilize as growth substrates the chlorinated benzoic acids produced from PCB breakdown (Field and Sierra-Alvarez, 2008). Thus, organisms such as the chlorobenzoate-degrader *Pseudomonas aeruginosa* JB2 (Hickey and Focht, 1990) can enhance PCB mineralization when growing alongside the PCB-cometabolizers (Hickey et al., 1993). It’s also possible to create more effective PCB-degraders by introducing into these organisms genes that encode chlorobenzoate metabolism. Generation of such hybrids could be greatly facilitated if genes of interest are naturally associated with MGE.

The *ohb* genes encoding degradation of a range of *ortho*-chlorobenzoates have been identified in *P. aeruginosa* JB2 (Hickey and Sabat, 2001) and demonstrated to transfer from *P. aeruginosa* JB2 to other bacteria (Perezlesher and Hickey, 1995; Hickey et al., 2001). Moreover, bacteria acquiring the *ohb* genes concomitantly acquired *clc* genes and *hyb* genes encoding for the metabolism of *ortho*-hydroxybenzoate (Hickey et al., 2001). The goal of this study was to identify MGE that are associated with these genes in the genome of *P. aeruginosa* JB2.

## Materials and Methods

### DNA Preparation

*Pseudomonas aeruginosa* JB2 was grown on 2-chlorobenzoate (2-CBa) as described previously (Hickey and Focht, 1990) and cells harvested in late log phase for use in genomic DNA preparations. Two approaches were used for genomic DNA (gDNA) preparation from *P. aeruginosa* JB2 that were compatible with the sequencing technology applied. For use in short read sequencing (Illumina 250 HiSeq) and long-read sequencing by single molecule, real-time sequencing (SMRT) using PacBio technology, gDNA was prepared by using an Illustra Bacterial DNA Minispin kit (GE Healthcare) following the manufacturer’s suggested protocol. For nanopore DNA strand sequencing (Oxford Nanopore Technologies, ONT), DNA was extracted from approximately 5 × 10^6^ cells of *P. aeruginosa* by using the MagAttract HMW DNA Kit (Qiagen) following the manufacturer’s instructions. The quality of all gDNA preparations was assessed with a Qubit fluorometer (Invitrogen) to quantify nucleic acid concentration, and with a Fragment Analyzer (Advanced Analytical Technologies, Inc.) using the High Sensitivity Large Fragment 50 Kb Analysis Kit to determine the fragment size distribution.

### Library Preparation and Sequencing

The PacBio sequencing library was prepared following the Accel-NGS XL Library Kit for PacBio protocol (Swift Biosciences). The resulting library was size-selected to 20 kb with a PippenHT (Sage Science). A final library QC was performed with a Qubit fluorometer to quantify library concentration and fragment size distribution of the library was determined on a Fragment Analyzer (Advanced Analytical Technologies, Inc.) using the High Sensitivity Large Fragment 50 kb Analysis Kit. The prepared sequence library was loaded onto a single Sequel v 2.1 SMRTcell at a concentration of 6 pM, following the PacBio diffusion loading protocol and including a polymerase-bound complex cleanup. One 600-min movie was taken of the SMRTcell. Illumina sequencing was done with an Illumina regular fragment library.

For ONT sequencing, *ca.* 1,500 ng of gDNA was used as input for the 1D genomic DNA by ligation (SQK-LSK108) protocol version GDE_9002_v108_revT_18Oct2016 (ONT). This preparative step included a DNA repair procedure to repair nicks, an end-repair step that also included dA-tailing of double-stranded DNA, ligation of sequencing adapters, AMPure XP bead purification, and tether protein attachment. Sequencing was performed as recommended by the manufacturer’s guidelines using R9.4 flow cells (FLO-MIN106). MinION sequencing was controlled with MinKNOW software (v 18.01.6; ONT). Data was collected for approximately 26 h. Base-calling was performed with Albacore (v 2.1.7; ONT). Only those reads meeting a minimum quality threshold, as determined by Albacore, were used for downstream analyses.

### Genome Assembly

Canu v1.7 (c9ef921) was used with PacBio sequencing reads >2 kb to assemble an initial 40-fold coverage. Canu was run with default parameters in grid mode (Sun Grid Engine) but with an estimated genome size of 6.8 Mb, a value based on other sequenced strains of *P. aeruginosa*. The completed assembly resulted in two closed (circular) contigs, one 6,822,869 bp, and another of 22,190 bp. Automated genome annotation with Prokka (Seemann, 2014) identified in the larger contig the replication initiation factor *dnaA*, which promotes the unwinding of DNA at *oriC*. However, the smaller contig lacked an origin of replication, and contained only the *ohb* and *hyb* clusters along with uncharacterized genes and insertion sequence elements.

The characteristics of the small contig suggested the presence of a repeat, which may have been collapsed into a single copy during assembly. To test this hypothesis, BLAST-N (v. 2.6.0) was applied in homology searches of the Canu MHAP corrected reads using selected genes from the *ohb* and *hyb* clusters (*ohbB, hybB*), and the region adjoining the *hyb* cluster (*mhqB*). Although the N50 of filtered, preassembly, subread lengths was approximately 14 kb, a small percentage of corrected reads extended beyond 24 kb. Some of these longer reads contained *ohbB, hybB* and *mhqB*. Approximately 7.5 kb beyond the stop codon of *ohbB*, another region with near exact homology to *hybB* was observed, followed by the beginning sequence of *mhqB* approximately 8.3 kb further. While this evidence was consistent with our hypothesis, none of the PacBio reads were sufficiently long to verify a complete repeat consisting of the three genes.

Guided by the premise that read length is an important determinant of genome assembly contiguity, we performed the same BLAST-N procedure using reads collected from the MinION sequencer. The N50 read length from the nanopore device was approximately 36.5 kb with many additional reads extending beyond 120 kb. With this long-read information, we were able to confirm a repeat that contained *ohbB, hybB* and *mhqB*, and spanned approximately 36 kb. To integrate this repeat into the large contig assembly, 40 of the highest quality MinION reads containing the complete repeat were aligned to the Canu assembly with Graphmap (Sovic et al., 2016) with the -C option enabled, to resolve coverage drops near the genome ends, should reads map near the ends. Racon (Vaser et al., 2017) was then used with uncorrected PacBio reads to generate a consensus and polish the entire assembly, including the repeated region.

The presence of the repeat and its position in the consensus assembly was confirmed with nucmer in the MUMmer-3.23 package (Kurtz et al., 2004). Lastly, paired-end, short-read Illumina data (approximately 51X coverage) were aligned to the consensus with bwa mem v0.7.12-r1039 (Li, 2013). Two rounds of polishing with Pilon v1.22 (Walker et al., 2014) were then applied to correct single base differences and small insertion and deletion events missed by consensus calling steps in Canu and Racon. This final polishing step resulted in 881 changes, reflecting a pre-correction agreement of 99.72 percent.

### Comparative Structural Analysis of Wild Type and Mutant ICEclc-JB2 by PCR

A spontaneous mutant of *P. aeruginosa* JB2 deficient in growth on 2-CBa was acquired by culturing on 1% glycerol as described previously (Hickey and Focht, 1990). Genomic DNA was extracted from the mutant by using an Illustra Bacterial Genomic DNA kit (GE Healthcare). PCR primers were designed to target regions in ICEclc-JB2 that were diagnostic of structure in the variable and core key regions (**Table 1**). All oligonucleotides used as primers were confirmed by BLAST against the *P. aeruginosa* JB2 genome as specific for the targeted location. PCR was done with *ca*. 50 ng of gDNA template in OneTaq Hot Start Master Mix containing Standard Reaction Buffer (New England Biolabs) according to the manufacturer’s recommendation. The thermal cycling program was an initial denaturation (94°C, 30 s) followed by 30 cycles of: 94°C (15 s), 55°C or 62°C (15 s) and 68°C (60 s). The final extension was 68°C for 5 min. The annealing temperature used in Step 2 of the 30 cycle program was either 55°C or 62°C depending on the primer set (**Table 1**). All PCR assays were run in an Eppendorf Mastercycler Nexus Thermal Cycler. Analysis of PCR products was done by electrophoresis in agarose gels (1% in TAE buffer) at 100 V for 3 h. The size of PCR products was assessed by comparison to a GeneRuler 1 kb DNA ladder (ThermoFisher).

**Table 1:** PCR Primers used for deletion mapping^a^.

Set	Sequence (5′-3′)	T_m_ (°C)
1	AGATTCTTGGGCGCTGTATC	55
1	GGTTGATCAGTTCGTTGCAATAG	55
2	CTCACCAGTGGCGTCAATAA	55
2	GCATACGTCAGCAAGGATCA	55
3	CGATCTCGGCGTCAAAGATT	55
3	GGTAGCGCTTGTTCAGGTATAG	55
4	TACCACCAGTGGGACTACAA	55
4	CAGGTTCGGGAAGATCGAAAG	55
5	CCACCGTTTGATGTTGGATTAG	62
5	GAAGTAGTTCGACCCACCTATG	62
6	CCTACACCGACAAAGACATCTAC	55
6	TGGCTACCTTCAGCTTGTTC	55
7	TGCGAAGGTCTCTCCTTTCTA	62
7	CCAAGCCCTCAGTTCGTTAAG	62
8	CGATATCGCAGTCAGGAGAGA	62
8	CTTCAGGCCATGGAAGACTATATG	62
9	CAGCGCGATCAATGCAATAG	62
9	GTCGCCAACATGCTCAATATC	62
10	TCCTGAACAGCACCATCATC	62
10	CAGTCATCGTCCACCCATTT	62
11	GATTCTGCAAATCTGTCTCGGTA	62
11	CTAGGGCAGTAAGTCGTTGATT	62

*^*a*^Set number refers to primer binding site locations shown in **Figure 3A**.*

### Nucleotide Sequence Deposition

The complete genome of *P. aeruginosa* JB2 is deposited in the National Center for Biotechnology Information Genbank database under accession number CP028917.1.

## Results and Discussion

### Overview of ICEclc-JB2 Structure and Comparisons to Other ICEclc

In *P. aeruginosa* JB2, ICEclc (hereafter referred to as ICEclc-JB2) is located between bases 4,860,575 to 4,983,845 (123,270 bp) and is delimited by repeated tRNA^Gly^ genes (76 bp). This structure of genomic integration is similar to that of ICEclc-LB400, which is also bounded by repeated tRNA^Gly^ genes (79 bp). In contrast, only one end of ICEclc-B13 is bounded by a full tRNA^Gly^ gene and the other end is delimited by a repeat of the last 18 bp of the tRNA^Gly^ gene. The *P. aeruginosa* JB2 genome possess a single copy of ICEclc-JB2, in contrast to ICEclc-B13, which is present in two copies in the genome of *P. knackmussii* B13 (Miyazaki et al., 2015). Within the genome of strain JB2, all genes contained with ICEclc-JB2 were present only within that element.

**FIGURE 1:**
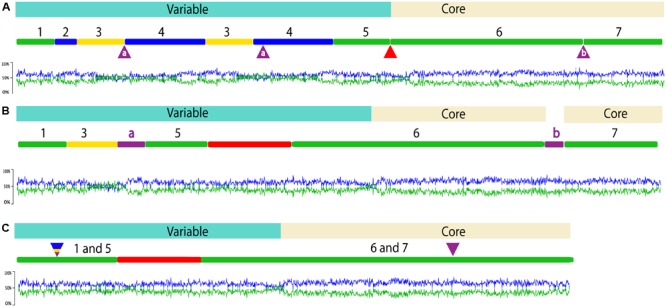
Overview of ICEclc-JB2 structure **(A)** and comparison to ICEclc-LB400 **(B)** and ICEclc-B13 **(C)**. In each panel, the element length is indicated by the numbered line and regions carrying cargo genes and core genes are shaded blue and beige, respectively. Graphs below each element display the %G + C skew. For ICEclc-JB2, green regions are conserved with both ICEclc-LB400 and ICEclc-B13, yellow regions are shared with ICEclc-LB400 only and blue regions are not shared with either ICEclc-LB400 or ICEclc-B13. The numbers across the top of these regions in ICEclc-JB2 are used to identify their locations in ICEclc-LB400 and ICEclc-B13. In ICEclc-JB2, the red triangle between regions 5 and 6 indicates the location of the *amp* cluster that is present in ICEclc-LB400 and ICEclc-B13 (red region, **B,C**), but absent in ICEclc-JB2. The purple triangles between regions 3 and 4, and between regions 6 and 7 indicate the locations of sequence occurring only in ICEclc-LB400 (purple bars labeled “a” and “b”; **B**). For ICEclc-B13 **(C)** the multicolor triangle indicates the location at which regions occur in ICEclc-JB2 and/or ICEclc-LB400, but are absent from ICEclc-B13. The purple triangle above ICEclc-B13 regions 6 and 7 indicates the location of sequence occurring only in ICEclc-LB400 (purple bar labeled “b”; **B**). The sizes of the elements presented are: 123,270 bp (ICEclc-JB2), 122,836 bp (ICEclc-LB400) and 105,032 bp (ICEclc-B13).

ICEclc-JB2 exhibits the biomodal structure that is characteristic of ICEclc-B13 and ICEclc-LB400 with variable (cargo) genes encoding biodegradation functions segregated to one end, followed by *ca*. 52 kb of core genes conserved within all three ICEclc (**Figure 1**). For ICEclc-B13 and ICEclc-LB400, the cluster of genes encoding aminophenol catabolism (*amp*) is a major component of the cargo area (29,300 bp), but is absent in ICEclc-JB2 (**Figure 1**). Two groups of cargo genes possessed by all three ICEclc are the cluster of genes annotated as encoding an anthranilate dioxygenase (Gaillard et al., 2006) immediately downstream of *intB13* (**Figure 1**, Region 1), and the *clc* genes (**Figure 1**, Region 5). The latter of these are well-characterized as encoding enzymes of the catechol *ortho*-cleavage pathway (*clcABCDE*) and a LysR element (*clcR*), which regulates expression of the *clc* operon (Ghosal and You, 1989). In contrast, the function of the putative anthranilate dioxygenase has yet to be determined, as none of the organisms possessing ICEclc have been reported to utilize anthranilate as a growth substrate.

**FIGURE 2:**
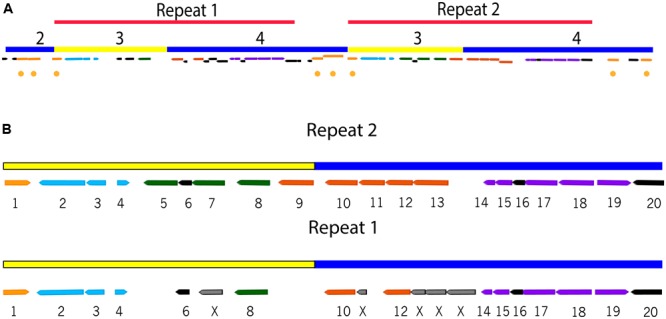
Repeat regions in the ICEclc-JB2 cargo area. **(A)** Overview of the location and orientation of the repeat regions within ICEclc-JB2 cargo area. The color-coding and numbering of bars is as described in **Figure 1**. The locations of transposases/insertion sequence elements are indicated by gold circles. **(B)** Detail of the repeat regions showing gene structure, numbering across the top of each repeat is the base pair location in the *P. aeruginosa* JB2 genome. Genes are numbered 1–20 based on structure in Repeat 2. Color-coding of genes is as follows: predicted transposase (gold; gene 1), *ohbBAR* (light blue; genes 2–4, respectively), predicted catabolic enzymes (green; genes 5, 7, 8), *hybIHGEF* (orange, genes 9-13, respectively), *hybDCABR* (purple; genes 14, 15, 17–19 respectively), hypothetical (black; genes 6, 16, 20). In Repeat 1, frame shifts result in the absence of genes 5 and 9 and the replacement of genes 7, 11, and 13 with fragmented ORFs (indicated by “X”). In **(B)**, Repeat 2 spans (left to right) genome positions 4,949,315–4,933,315 and Repeat 1 spans genome positions 4,971,315–4,955,315 (left to right).

The variable region of ICEclc-JB2 is distinct from that of other ICEclc in that its major block of biodegradation functions is present in a tandem repeat (**Figures 1, 2**). Each of the repeats contains the *ohb* and *hyb* genes (**Figure 2**), the former of these is shared with ICEclc-LB400 while the latter is not present in any other ICEclc yet reported. While the nucleotide sequence of the repeats is 99.9% identical, a number of indels occur in Repeat 1 some of which result in apparent frame-shifts and loss of several genes in Repeat 1 (**Figure 2**).

An element closely related to ICEclc, termed ICEXTD, has recently been characterized in several species and strains of *Azoarcus* (Zamarro et al., 2016). The close connection of ICEXTD to ICEclc is based on synteny of core functions (Zamarro et al., 2016). Also, like ICEclc, ICEXTD integrates at tRNA^Gly^ genes, although it’s attachment site spans the terminal 23-bp instead of 18 bp as for ICEclc-B13 (Zamarro et al., 2016). Ecologically, ICEXTD plays a role similar to that of ICEclc, and endows upon the host the ability to utilize aromatic compounds as growth substrates (Zamarro et al., 2016). For ICEXTD, these compounds are toluene, *m*-xylene and cumene, the latter two of which support aerobic and anaerobic growth (Zamarro et al., 2016). While ICEXTD exhibits ecological and evolutionary similarities to ICEclc, ICEXTD differs significantly from ICEclc in structure in two respects. First, the core genes and cargo (termed adaption modules) are intermixed in ICEXTD, organization of core and cargo regions varies between ICEXTD that originate from different strains or species (Zamarro et al., 2016).

### The *ohb* Cluster of ICEclc-JB2

Key growth substrates for *P. aeruginosa* JB2 are 2-CBa, 2,4-dichlorobenzoate, 2,5-dichlorobenzoate, and 2,3,5-trichlorobenzoate, which are transformed by the *ohbAB-*encoded dioxygenase to catechol, 4-chlorocatechol (2,4- and 2,5-dichlorobenzoate) and 3,5-dichlorocatechol, respectively (Hickey and Sabat, 2001). Prior work established that transformation of all *ortho*-chlorobenzoates and chlorocatechols is induced by growth on *ortho*-chlorobenzoates (Hickey and Focht, 1990). The *clc* cluster that occurs on ICEclc-JB2 encodes the enzymes adapted for funneling the chlorocatechols produced by the activity of OhbAB into pathways of central metabolism. Thus, physical linkage of *ohb* and *clc* within ICEclc-JB2 makes the element a potentially efficient vehicle for conferring on a host the ability to utilize *o*-halobenzoates as carbon sources. Initial work with *P. knackmussii* B13 illustrated the importance of ICE*clc* in establishing the ability for utilization of 3-chlorobenzoate (Reineke and Knackmuss, 1980). But, in this case, the initial step of 3-chlorobenzoate transformation to 3-chlorocatechol was mediated by a dioxygenase that was not present within ICEclc-B13, but was instead encoded by an *xylXYZ* ortholog located elsewhere in the *P. knackmussii* B13 genome (Miyazaki et al., 2015).

To date, only three records exist in Genbank for *ohbAB* orthologs in organisms other than *P. aeruginosa* JB2: *Achromobacter xylosoxidans* A8 (CP002288, pA8-1), *Burkholderia xenovorans* LB400 (CP000270) and *Pseudomonas aeruginosa* 142 (AF121870). With the exception of *P. aeruginosa* 142, these records collectively establish that association with a self-transmissible MGE is a common feature of the *ohb* cluster although the nature of the element is variable: ICEclc in the case of *P. aeruginosa* JB2 and *B. xenovorans* LB400 and a 98 kb IncP1-β plasmid in the case of *A. xylosoxidans* A8. For *P. aeruginosa* 142, an unequivocal assessment of association with an MGE is not possible as the current data for strain 142 is limited to a partial record of 6,052 bp that encompasses only the *ohb* genes and flanking regions. However, based on what is known about *ohb* in the other organisms discussed here, association of *ohb* with ICEclc or other MGE might be expected for *P. aeruginosa* 142.

The *ohb* clusters are similar in that *ohbA* is immediately adjoined downstream by a divergently oriented coding sequence (*ohbR*) for which an IclR-type transcriptional regulatory element is predicted. For ICEclc-JB2, ICEclc-LB400 and pA81, *ohbR* is 255 bp with a predicted polypeptide of 85 amino acids. In contrast, for *P. aeruginosa* 142, *ohbR* is 717 bp with a predicted polypeptide of 239 amino acids. The predicted polypeptides of both the long and short versions of *ohbR* possess the N-terminal LTTR motif characteristic of IclR elements, which is required for recognition of DNA binding sites (Tropel and van der Meer, 2004). However, IclR function also requires C-terminal structure essential for the binding of effector molecules and for the multimerization of IclR polypeptides to tetramers, the supramolecular structure active in binding DNA (Tropel and van der Meer, 2004). Thus, while regulation of *ohbAB* has not been empirically determined for any organism, it appears that the predicted OhbR of *P. aeruginosa* 142 could play a role in this process, as it possesses the N-terminal and C-terminal structure requisite for function as an IclR regulator. But, the apparently truncated version of OhbR predicted in the other three cases would not be functional according to criteria established for IclR elements (Tropel and van der Meer, 2004). While the genome of strain JB2 has only one copy of *ohbR*, it possesses numerous other predicted IclR elements that could affect *ohbAB* expression; empirical research is needed to delineate the regulatory system for *ohbAB*.

Of the four organisms that possess *ohbAB, B. xenovorans* LB400 is the only one for which growth on *o*-chlorobenzoates (or any chlorobenzoic acid) has not yet been demonstrated. The novel phenotype of *B. xenovorans* LB400 [*a.k.a. Pseudomonas* sp. LB400 (Bopp, 1986), *Burkholderia* sp. LB400 (Bartels et al., 1999), *Burkholderia fungorum* LB400 (Marx et al., 2004), *Paraburkholderia xenovorans* LB400, (Sawana et al., 2014)] is a relatively broad spectrum of PCB congeners that it transforms by cometabolism (i.e., growth on biphenyl) to a variety of products. Cometabolism by *B. xenovorans* LB400 of some lower chlorinated PCBs yields chlorobenzoic acids, but strain LB400 does not grow on chlorobenzoic acids, which limits growth on PCBs (Potrawfke et al., 1998). In particular, specific testing with 2-CBa as a sole carbon source failed to elicit growth by *B. xenovorans* LB400 (Rodrigues et al., 2006). It should be noted that these studies pre-dated genome sequencing of *B. xenovorans* LB400 (Chain et al., 2006), and the existence of ICEclc-LB400 (and the *ohb* genes) in *B. xenovorans* LB400 was unknown. Thus, in an effort to improve the PCB-degradation capabilities of *B. xenovorans* LB400 by transforming it to a strain that could grow *o*-chlorobenzoates (and thus grow on some PCBs), *ohbRAB* were cloned from *P. aeruginosa* 142 (Tsoi et al., 1999) and introduced to *B. xenovorans* LB400, yielding a transformant designated as *B. xenovorans* LB400(*ohb*) that was capable of growth with 2-CBa (Rodrigues et al., 2006).

It’s unclear why introduction of *ohbRAB* to *B. xenovorans* LB400, an organism that natively possessed the *ohb* genes, was necessary to enable its growth on 2-CBa. Possibly, the full-length *ohbR* introduced with the *P. aeruginosa* 142 *ohb* cluster complemented the truncated *ohbR* native to ICEclc-B13, and thereby provided a regulatory function that alleviated a restriction on gene expression. But, if so, that would mean that two different organisms (*P. aeruginosa* JB2 and *A. xylosoxidans* A8) that also possess a truncated *ohbR*, yet grow on 2-CBa, can complement the function of the truncated *ohbR*, whereas *B. xenovorans* LB400 cannot. While its unknown why *P. aeruginosa* JB2, *A. xylosoxidans* A8 and *B. xenovorans* LB400 differ in the utilization of 2-CBa, the divergent phenotypes of these three organisms underscore the importance of the host background as a modulator of the expression of functions encoded by ICEclc specifically, and MGE in general.

The physical linkage of genes encoding *o*-chloro-benzobenzoate (*ohbAB*) and *o*-hydroxybenzoate (*hybABCD*) metabolism was implied in previous studies, wherein these phenotypes were acquired simultaneously by bacteria mated with *P. aeruginosa* JB2 (Hickey et al., 2001). The present description of ICEclc-JB2 provides insight into the associations with MGE that may have been involved in those gene transfers. The *hyb* region (**Figure 2**) includes a LysR regulatory element (*hybR*), a ring-hydroxylating monooxygenase (*hybABCD*, transforms salicylate to gentisate), and the components of an ABC-type uptake (*hybEFGHI*). The *hyb* cluster is distinct in exhibiting a %G + C content that is significantly lower than that of the neighboring *ohb* genes indicating an origin divergent from that of *ohb* (**Figure 1**).

### ICEclc-JB2 Lacks Genes Encoding Key Regulatory Functions

Functions have been identified within the core region of ICEclc-B13, which control its stabilization and mobilization (Minoia et al., 2008; Miyazaki and van der Meer, 2011; Pradervand et al., 2014; Delavat et al., 2016). A key feature is an operon of three genes that effects global regulation of transfer initiation (Pradervand et al., 2014). These genes are designated as *tciR, marR, mfsR*, and encode a LysR-type activator (TciR), a MarR-type regulator (MarR) and a TetR-type repressor (MfsR), respectively. The *tciR-marR-mfsR* operon also exists in ICEclc-LB400 and is located at same position as in ICEclc-B13, the junction between the *clc* region (Region 5, **Figure 1**) and the *amp* cluster (red segment, **Figure 1**). However, this location is contained with a *ca*. 28 kb region of ICEclc-B13/LB400 that is absent in ICEclc-JB2, which corresponds to the region spanning ORF18502 to ORF4677 in ICEclc-B13 (Gaillard et al., 2006). The *tciR-marR-mfsR* genes do not exist elsewhere in the strain JB2 genome. The absence of *tciR-marR-mfsR* from ICEclc-JB2 indicates that either other genes are the functional equivalent of this operon and/or the regulation of ICEclc-JB2 transfer occurs by mechanisms different from those established for ICEclc-B13. Identification of additional ICEclc relatives is needed to determine the frequency with which the *tciR-marR-mfsR* cluster is present or absent from these elements. For ICEclc-JB2, and other ICEclc relatives lacking *tciR-marR-mfsR*, studies are needed to elucidate the systems that control stabilization and mobilization of the elements.

### Independent Mobilization of ICEclc-JB2 Cargo Genes

Earlier studies with *P. aeruginosa* JB2 demonstrated that the spontaneous loss of the 2-CBa utilization phenotype occurred when strain was cultured on a substrate such as glycerol (Hickey and Focht, 1990). The data presented in this report supports two hypotheses about the process(es) that gave rise to the mutant phenotype. Given that ICEclc controls its own excision, one hypothesis is that the entire element is lost from the genome. Alternatively, the repeats harboring the *ohb* genes are neighbored by multiple transposases/insertion sequence elements (**Figure 2A**) and its possible *ohb* loss results from excision of these elements. In this case, elimination of *ohb* would occur independently of ICEclc-JB2 as a whole, which would remain in the genome absent the *ohb* genes.

**FIGURE 3:**
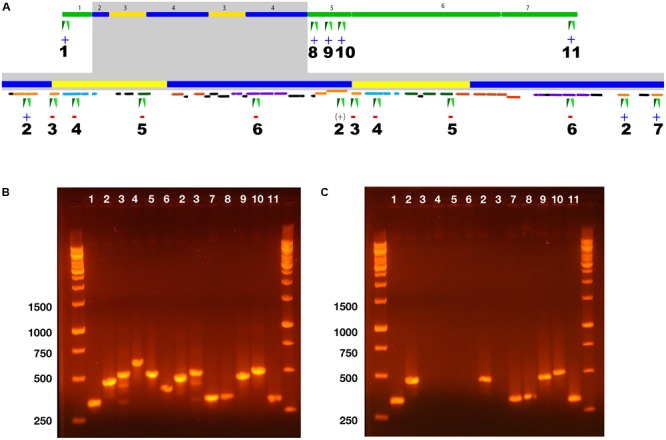
PCR Analysis of ICEclc-JB2 structure in wild type *P. aeruginosa* JB2 and a spontaneous mutant of strain JB2 deficient in growth on 2-CBa. **(A)** Map of ICEclc-JB2 displaying locations of 11 PCR primer pairs used to probe its structure. The gray box indicates expanded detail of the repeat region. Color-coding of genes follows is as used in **Figure 2**. The symbols “+” or “–“ beneath numbers 1–11 indicate the presence or absence, respectively of a PCR product in the mutant for the indicated primer pair. For primer pair 2, the symbol “(+)” is placed beneath its location between repeats 1 and 2 to indicate that the product observed in the mutant was likely derived from one or both of its other two binding sites that are located at the termini of the repeats. **(B)** Agarose gel of PCR products from the wild type. **(C)** Agarose gel of PCR products from the mutant. Numbering across the top of each gel corresponds to the primer pair location indicated in **(A)**. Numbering along the left side of gels indicates the size of the five bottom bands in the DNA ladder.

To test these two hypotheses, a 2-CBa-deficient mutant (derived from a glycerol-grown culture) was examined by PCR to determine the presence/absence of regions located in ICEclc core regions, genes within the repeats and transposable elements that border the repeats (**Figure 3**). The wild type and mutant both gave expected PCR products for regions targeting either end of ICEclc-JB2 (**Figure 3**, primer sets 1, 11) and to primers targeting *clc* genes in the conserved region adjacent to the repeat region (**Figure 3**, primer sets 8–10). The mutant was also positive in PCR for primer pair 2, which targeted an insertion sequence element that was present at either end of the repeat region (**Figure 3**). In contrast, primers targeting *ohbB, hybB* and a gene between *ohbB* and *hybB* (gene 8, **Figure 2B**) were all negative in the mutant (**Figure 3**, primer sets 4–6). Thus, the boundaries of the excised region lay between the insertion sequence element targeted by primer pair 2 (bases 4,930,538 – 4,974,802).

Collectively, results of the PCR analysis support the hypothesis that the mutant phenotype resulted from loss of the *ohb* genes from ICEclc-JB2, not loss of ICEclc-JB2 as a whole. Loss of the *ohb* genes was associated with excision of the entire repeat region that encompassed a total of *ca*. 44 kb. Thus, more than half of the cargo region can excise independently of ICEclc-JB2, and possibly involves the transposases/insertion sequence elements that are associated with the repeat region. Loss of the repeat results in the loss of key, selective phenotypes encoded by ICEclc. In the short term, loss of the repeat region potentially makes it less useful to a host. However, the reduced version of ICEclc-JB2 would now have much unused physical capacity for cargo, which it could use to gain new genes (phenotypes) that might be beneficial to a future host and, consequently, to the long-term survival of ICEclc.

## Conclusion

The present report establishes a new ICE, ICEclc-JB2, which is closely related to ICEclc-B13 and ICEclc-LB400. All three elements are ecologically similar in that they potentially confer on the host biodegradation phenotypes. For ICEclc-JB2 unique the characteristics endowed are the utilization of *o*-halobenzoates and *o*-hydroxybenzoate as growth substrates. While ICEclc-JB2 is highly similar to ICEclc-B13 and ICEclc-LB400, it lacks key a regulatory gene that is present in the other two ICEclc, which controls excision from the host. Thus, the mechanisms regulating intracellular behavior of ICEclc-JB2 may differ from that of its close relatives. More than half of the cargo genes carried by ICEclc-JB2 can excise independently from the element, potentially providing evolutionary flexibility for the element.

## Author Contributions

CO, SV, VG, MB, JW, DP, JH, and WH all contributed to experimental work, data analysis, and preparation of the manuscript.

## Conflict of Interest Statement

The authors declare that the research was conducted in the absence of any commercial or financial relationships that could be construed as a potential conflict of interest.
